# Comprehensive review of male breast cancer: Understanding a rare condition

**DOI:** 10.32604/or.2025.058790

**Published:** 2025-05-29

**Authors:** ABDUR JAMIL, RIMSHA SIDDIQUE, FARYAL ALTAF, DANIYAL WARRAICH, FAIZAN AHMED, ZAHEER QURESHI

**Affiliations:** 1 Department of Medicine, Samaritan Medical Centre, Watertown, NY 13601, USA; 2 Independent Research Associate, Watertown, NY 13601, USA; 3 Department of Internal Medicine, Icahn School of Medicine at Mount Sinai/BronxCare Health System, New York, NY 10457, USA; 4 Department of Internal Medicine, Sahiwal Medical College, Sahiwal, 57040, Pakistan; 5 Division of Cardiology, Duke University Hospital, Durham, NC 27710, USA; 6 Department of Internal Medicine, School of Medicine at Quinnipiac University, Bridgeport, CT 06473, USA

**Keywords:** Male breast cancer (MBC), Survival analysis, Genetic mutations, Hormone therapy, Targeted therapy, BRCA mutations, Hormonal imbalance

## Abstract

**Background:**

Male breast cancer (MBC) is a rare but significant health concern, accounting for less than 1% of all breast cancer cases. Despite its low incidence, it presents unique clinical, genetic, and psychosocial challenges. Genetic predispositions, including BRCA2 mutations and hormonal imbalances, are key factors influencing the development of MBC. However, the rarity of the condition has led to limited research and fewer treatment guidelines specifically for male patients.

**Methods:**

A comprehensive literature review was conducted using PubMed, MEDLINE, and Embase databases to identify studies focusing on the epidemiology, risk factors, clinical presentation, diagnosis, treatment, and psychosocial impacts of male breast cancer. Articles were selected based on relevance, peer-review status, and focus on MBC in male patients. Data were synthesized narratively, and findings were contextualized based on the methodology and design of included studies.

**Results:**

The review identified several significant risk factors for MBC, including BRCA2 mutations, hormonal imbalances (particularly estrogen and testosterone levels), and family history of breast cancer. MBC is often diagnosed at later stages due to the absence of routine screening in men, resulting in poorer survival outcomes compared to female breast cancer. Treatment strategies for MBC largely mirror those for women, including surgery, radiation, chemotherapy, and hormonal therapies. However, the psychosocial impacts of MBC are unique to men, with issues such as stigma, body image concerns, and societal perceptions of masculinity.

**Conclusions:**

Male breast cancer remains an understudied area of oncology, with significant gaps in research related to early detection, targeted therapies, and long-term care. Collaborative international research efforts, such as the MERGE consortium and the International Male Breast Cancer Program, are essential for improving understanding and treatment outcomes. Genetic counseling, early screening, and personalized treatment approaches are crucial in managing the disease. Further research focusing on the molecular basis of MBC, along with the psychosocial needs of affected men, is necessary to enhance both survival rates and quality of life for male breast cancer patients.

## Introduction

Breast cancer is commonly thought to affect women primarily, but it is crucial to understand that men can also be affected by this disease [1]. While male breast cancer is uncommon, it is a significant health concern that deserves thorough investigation and heightened awareness [2]. Even though male breast cancer is rare, it should not minimize the significance of comprehending and effectively addressing this disease, given its profound physical and psychological impacts on those affected [3].

Male breast cancer is scarce, making up less than 1% of all breast cancer cases worldwide and about 0.2% of all male cancers. According to global cancer statistics, the incidence of male breast cancer varies across different regions and populations [4]. In the United States, the estimated incidence rate is approximately 1.3 cases per 100,000 men per year [4]. In contrast, the incidence rate in women is significantly higher, with approximately 284 cases per 100,000 women per year [4]. This difference in incidence rates between men and women highlights the rarity of male breast cancer. Thailand had the lowest male breast cancer incidence rate at 0.16 per 100,000 person-years, followed by Japan, Singapore, and Colombia [5]. However, despite its low incidence, it represents a significant health concern that warrants attention and thorough investigation. Research evidence shows that there has been a gradual increase in incidence rates of male breast cancer from the 1980s to the 2000s [6,7], highlighting the growing prevalence of this rare condition. Epidemiology and Demographics of Male Breast Cancer are shown in Table 1.

**Table 1:** Epidemiology and demographics of male breast cancer

Characteristic	Value	Reference
Incidence	<1% of all breast cancer cases worldwide	[8,9]
	Increased from 7.2% in 2004 to 10.3% in 2014 in the US	[10]
Race/Ethnicity	75% Non-Hispanic White	[9]
	12% Non-Hispanic Black	[9]
	0.5% Hispanic	[9]
Age at diagnosis	Mean age: 64.6 ± 13.1 years	[9]
Mortality rates	Highest in Non-Hispanic Blacks (29%)	[9]
	Non-Hispanic Whites (27%)	[9]
	Hispanics (21%)	[9]
Tumor stage	32.1% Stage I	[10]
	34.6% Stage II	[10]
Tumor grade	84.5% Moderate or poorly differentiated	[10]
Hormone receptor status	90% ER-positive	[10]
	81% PR-positive	[10]
HER2 status	Lower HER2 overexpression compared to females	[10]

Clinically, male breast cancer presents as a painless subareolar mass with nipple ulcerations and retraction presenting as signs of cancer [11]. Research evidence across the oncology field has suggested the presentation of these cancer signs at varying timelines, with a Spanish study averaging the onset and diagnosis of the symptoms at ten months [12]. Based on assertions made by Korde et al. [13], male breast cancer is likely to be diagnosed at a later stage compared to female breast cancer. The study attributes this assertion to the lack of awareness of male breast cancer presentation among the general population [13]. Thus, several factors have been attributed to the limited literature on male breast cancer, including its rarity, stigma (treatment and management techniques for male breast cancer are mainly based on those for women), and low levels of awareness within the general population [14–16].

This literature review aims to comprehensively synthesize the current knowledge and research findings on male breast cancer. By highlighting the epidemiological trends, risk factors, diagnostic approaches, treatment modalities, and unique challenges male patients face, this review seeks to raise awareness and promote a deeper understanding of this rare but significant disease entity.

## Methods

### Literature search strategy

A comprehensive literature search was conducted to identify relevant studies on male breast cancer (MBC). The search used PubMed, MEDLINE, and Embase databases from their inception until July 2024. Keywords and Medical Subject Headings (MeSH) terms used included “male breast cancer,” “gynecomastia,” “treatment,” “diagnosis,” “prognosis,” and “epidemiology.” Boolean operators (AND, OR) were applied to combine search terms. Additionally, references of selected studies were manually reviewed to capture any potentially missed articles.

### Inclusion and exclusion criteria

Studies were selected based on the following inclusion criteria: 1. Focus on male breast cancer. 2. Publication in peer-reviewed journals. 3. Provision of data on epidemiology, risk factors, clinical presentation, pathology, treatment strategies, prognosis, or psychosocial impacts. 4. Articles written in English.

Studies were excluded based on the following exclusion criteria: 1. Studies not published in English. 2. Preclinical or animal studies. 3. Studies needing more detailed methodology or results.

### Study selection

The selection process involved two independent reviewers who screened the titles and abstracts of all identified articles. They then retrieved the full texts of potentially relevant studies and evaluated them for eligibility based on the inclusion and exclusion criteria. Discrepancies between reviewers were resolved through discussion, and a third reviewer was consulted if necessary to reach a consensus.

### Data extraction and synthesis

The extracted data were synthesized narratively, emphasizing key themes and patterns related to male breast cancer. Where applicable, quantitative data were summarized in tables to aid in comparison and interpretation.

### Quality assessment

Due to the narrative nature of this review, a formal systematic quality assessment was not conducted. However, studies were categorized based on their design (e.g., randomized controlled trials, observational studies) and the robustness of their methodologies to provide context for the findings.

### Data presentation

The findings from the included studies were presented in a narrative format, focusing on the epidemiology, risk factors, clinical presentation, pathology, treatment strategies, prognosis, and psychosocial impacts of male breast cancer. Key outcomes were highlighted and discussed, with tables summarizing quantitative data and facilitating cross-study comparison.

### Ethical considerations

This narrative review utilized previously published data and did not involve primary data collection; therefore, ethical approval was not required. Ethical research conduct principles were followed, including proper citation and acknowledgment of original authors’ work.

### Male breast cancer epidemiology

Studies have shown that race and ethnicity influence male breast cancer median age diagnosis [10,17]. For instance, in the US, male breast cancer varies by nationality and race. Specifically, non-Hispanic men have a higher incidence rate, while Pacific and Asian men have the lowest rate [18]. Authors note that in 2021, the incidence rate annually among non-Hispanic black men was 2.2 per 100,000, 1.3 per 100,000 in non-Hispanic white men, 0.8 per 100,000 in non-Hispanic Pacific and Asian Islander men, and 0.7 per 100,000 in Hispanic men [18].

The mortality rate is also higher among non-Hispanic Black men compared to Hispanic and non-Hispanic white men [19]. In 2022, the research evidence establishes that the annual mortality rate was 0.6, 02, and 0.1 per 100,000 men among non-Hispanic black, non-Hispanic white, and Hispanic men, respectively [19].

### Clinical presentation and diagnosis

The clinical presentation of male breast cancer can vary widely, and symptoms may be initially ignored or attributed to other conditions, which can result in delays in diagnosis [20]. One of the most common presenting signs is a palpable mass or lump in the breast area, which may be painless or accompanied by discomfort, tenderness, skin changes, nipple retraction, or nipple discharge (which may be bloody or clear) [21,22]. Another significant symptom to note is the presence of swollen lymph nodes in the armpit (axilla) [23].

Diagnosing male breast cancer can be challenging due to the lack of routine breast screening programs for men and the potential for delayed recognition of symptoms [24]. Unlike women, who may undergo regular mammograms, men often do not seek medical attention until they notice a concerning lump or other breast changes [25]. This delay can lead to a more advanced stage at diagnosis, which can negatively impact treatment outcomes and prognosis [20].To confirm the diagnosis of male breast cancer, various imaging modalities and biopsy techniques are employed. Mammography, although less commonly used in men, can provide valuable information about the extent and characteristics of the breast lesion [26]. Ultrasound is often a complementary imaging technique, particularly for characterizing breast masses and guiding biopsy procedures [27,28]. Tissue biopsy through fine-needle aspiration or core needle biopsy is essential for confirming the diagnosis and determining the tumor’s histological subtype and molecular characteristics [29,30]. In some cases, additional imaging techniques, such as magnetic resonance imaging (MRI), may be used to evaluate the extent of the disease and aid in treatment planning [31]. Diagnostic techniques for Male Breast Cancer are shown in Table 2.

**Table 2:** Diagnostic techniques for male breast cancer

Diagnostic technique	Description	Advantages	Limitations
Mammography	X-ray imaging of the breast to detect abnormalities.	Widely available; Non-invasive; Suitable for detecting calcifications.	Less effective in dense breast tissue; Radiation exposure.
Ultrasound	Sound wave imaging to visualize breast tissue and detect abnormalities.	Suitable for distinguishing solids from cystic masses; Complementary to mammography.	Operator dependent may miss minor abnormalities.
MRI	Magnetic resonance imaging to provide detailed images of breast tissue.	Highly sensitive; Remarkably useful for dense breast tissue.	Expensive; Limited availability; May require contrast injection.
Fine Needle Aspiration (FNA)	Using a thin, hollow needle to remove small samples of tissue or fluid from a breast lump.	Minimally invasive; Quick procedure; Proper for initial diagnosis.	May not provide enough tissue for a definitive diagnosis.
Core needle biopsy	Using a more extensive, hollow needle to remove a core of tissue from a suspicious area.	Provides larger tissue samples; More accurate diagnosis than FNA.	More invasive than FNA; Potential for discomfort and bruising.
Excisional biopsy	Surgical removal of an entire lump or suspicious area for further analysis.	Provides a definitive diagnosis; Entire area can be analyzed.	Invasive; Requires local or general anesthesia; Longer recovery time.

Given the rarity of male breast cancer, healthcare professionals must maintain a high index of suspicion and promptly investigate any concerning breast symptoms in men [32,33]. Early detection and accurate diagnosis are critical for initiating appropriate treatment and improving outcomes for male breast cancer patients. Research shows that survival for patients with male breast cancer is similar to that for women with breast cancer when the diagnosis stage is the same [34].

### Pathology and molecular characteristics

Male breast cancer, like its female breast cancer, involves various histological subtypes with distinct molecular characteristics. The most common histological subtype is invasive ductal carcinoma, accounting for approximately 55%–85% of all male breast cancer cases [35–37]. Other subtypes, such as invasive lobular carcinoma, mucinous carcinoma, and papillary carcinoma, are less frequently encountered in men [35,37].

Molecular profiling of male breast cancer has revealed similarities and differences compared to female breast cancer. Like female breast cancer, male breast cancer can be classified into different molecular subtypes based on the expression of hormonal receptors (estrogen receptor [ER] and progesterone receptor [PR]) and the human epidermal growth factor receptor 2 (HER2) status [38].

Most male breast cancers are hormone receptor-positive, with approximately 80%–90% expressing ER and PR [6,39]. However, the proportion of HER2-positive cases in male breast cancer is lower compared to female breast cancer [40,41]. Additionally, male breast cancers tend to have a higher proportion of the luminal A-like subtype (ER+/PR+/HER2-) compared to female breast cancers [42].

Understanding the molecular characteristics of male breast cancer is crucial for guiding treatment decisions and developing targeted therapies [43]. While there are similarities with female breast cancer, the unique molecular landscape of male breast cancer underscores the need for customized research and therapeutic approaches.

### Risk factors and etiology

The etiology of male breast cancer is multifactorial, with hormonal factors playing a significant role in its pathogenesis. An imbalance between estrogen and testosterone levels can increase the risk of developing male breast cancer [44,45].

Estrogen exposure—either endogenous or exogenous—has been linked to an increased risk of male breast cancer [46]. Conditions leading to increased estrogen levels—like obesity and liver disease [47,48], along with exposure to exogenous sources of estrogens from medications or the environment—have been linked to a higher risk of male breast cancer [49].

Conversely, low levels of testosterone, the primary male sex hormone, may also contribute to the development of male breast cancer [50]. Testosterone is thought to have a protective effect against breast cancer in men [51]. Similarly, the hormone is protective against conditions or treatments that result in decreased testosterone levels, such as androgen deprivation therapy for prostate cancer or certain genetic disorders, which can increase the risk [51,52].

Besides, genetic mutations—particularly in the BRCA1 and BRCA2 genes—play a significant role in the development of male breast cancer [53]. For instance, men with BRCA2 mutations have a higher lifetime risk of developing breast cancer compared to those with BRCA1 mutations [54]. Other genetic syndromes, such as Klinefelter syndrome and Cowden syndrome, have also been associated with an increased risk of male breast cancer [55]. Additionally, family history increases the risk of male breast cancer, similar to female breast cancer [56]. A strong family history involves having a sister or mother with breast cancer, specifically if diagnosed at age 40 or younger. As a result, if one has a family member with breast cancer, it is advisable to perform genetic testing to determine whether they have a genetic mutation [56].

Environmental and lifestyle factors, such as radiation exposure, occupational hazards, alcohol consumption, and diet, have also been implicated in the etiology of male breast cancer [57]. However, the evidence is less intense compared to hormonal and genetic factors. Therefore, understanding the various risk factors and their interplay in the development of male breast cancer is crucial for identifying high-risk individuals, implementing preventive measures, and developing targeted screening and risk-reduction strategies. Risk factors of Male Breast Cancer are shown in Table 3.

**Table 3:** Risk factors for male breast cancer

Risk factor	Description	Reference
Age	Increased risk with advancing age. The median age for male breast cancer diagnosis varies by ethnicity and race. Specifically, male breast cancer diagnosis median age was 70, 66, 65, and 64 for non-Hispanic white, non-Hispanic Pacific and Asian Islander, non-Hispanic black, and Hispanic men, respectively.	[58–60]
Hormonal imbalance	Higher levels of estrogen relative to androgen hormones can occur due to obesity, testicular atrophy, liver disease, or Klinefelter syndrome.	[58,61]
Radiation exposure	Increased risk in men treated with radiation therapy to the chest area, e.g., for lymphoma.	[58,62,63]
Family history/Genetics	About 20% have a first-degree relative with breast cancer. Associated with BRCA2 mutations (accounts for ~10% of cases) and possibly CHEK2, PTEN, and PALB2 mutations.	[52,58,64]
Klinefelter syndrome	This is a rare genetic condition with an extra X chromosome, leading to hormonal imbalances and increased risk.	[58,65,66]
Obesity	High body mass index (BMI) is associated with 35% higher risk compared to low BMI.	[55,67,68]
Gynecomastia	The presence of excess breast tissue is associated with a 10-fold increased risk, independent of obesity and Klinefelter syndrome.	[68]
Alcohol consumption	Increased risk with higher alcohol intake.	[68]
Estrogen treatment	Prostate cancer treatment may increase breast cancer risk.	[55,58]

### Treatment strategies

The treatment strategies for male breast cancer essentially parallel those employed for female breast cancer, with some notable differences and considerations. The primary treatment modalities include surgery, radiation therapy, chemotherapy, hormonal therapy, and targeted therapies.

Surgery is typically the first line of treatment for male breast cancer, usually to remove the primary tumor and potentially involved lymph nodes [69]. Modified Radical Mastectomy, which involves the complete removal of the breast tissue, is the most common surgical approach for men [70]. However, in select cases, breast-conserving surgery (lumpectomy) may be considered, particularly for early-stage tumors [71]. The decision between mastectomy and lumpectomy depends on factors such as tumor size, location, and patient preferences [72].

Adjuvant therapies—chemotherapy, radiation therapy, and hormonal therapy—are often recommended following surgery to reduce the risk of recurrence and improve overall survival [73–75]. Chemotherapy regimens for male breast cancer are similar to those used in female breast cancer and are typically based on the tumor’s molecular subtype and stage [34,74]. Radiation therapy may be recommended after breast-conserving surgery or in cases of locally advanced or high-risk disease [34,73].

Hormonal therapy plays a crucial role in the treatment of hormone receptor-positive male breast cancer, which accounts for most cases [34,75]. Tamoxifen, an estrogen receptor modulator, is commonly used as adjuvant therapy or in the metastatic setting [76]. Other hormonal agents, such as aromatase inhibitors, may also be considered in some instances [77,78].

In recent years, targeted therapies have emerged as promising treatment options for male breast cancer patients [79]. For example, pertuzumab and trastuzumab, a monoclonal antibody targeting HER2, can be used in combination with chemotherapy for HER2-positive male breast cancers, similar to its use in female breast cancer [80].

While the overall treatment strategies for male breast cancer are primarily guided by the principles established for female breast cancer, it is essential to consider the unique anatomical and physiological differences between men and women [15]. Customized approaches and careful consideration of potential side effects, such as the impact on body image, sexual function, and masculinity, are essential in the management of male breast cancer patients [15]. Multidisciplinary care involving oncologists, surgeons, radiation oncologists, psychologists, and supportive care specialists is crucial to addressing the complex physical, emotional, and psychosocial needs of male breast cancer patients throughout their treatment journey [81]. Treatment approaches and outcomes for Male Breast Cancer are shown in Table 4.

**Table 4:** Treatment approaches and outcomes for male breast cancer

Treatment modality	Description	Commonly used drugs/Techniques	Outcomes	5-year survival rate (%)
Surgery	Surgical removal of the tumor, often through mastectomy.	Mastectomy, lumpectomy	High success rate in early stages; Risk of recurrence in later stages.	80%–90% (early stage), 50%–60% (late stage) [34,69]
Radiation therapy	Use of high-energy rays to target and kill cancer cells.	External beam radiation therapy	Effective in reducing local recurrence; Side effects include skin irritation and fatigue.	70%–80% [34,73]
Chemotherapy	Use of drugs to destroy cancer cells, typically given intravenously.	Anthracyclines, taxanes, cyclophosphamide	Improves survival rates; Side effects include nausea, hair loss, and increased infection risk.	60%–70% [34,74]
Hormone therapy	Use of hormone-blocking therapy, such as tamoxifen, to reduce hormone levels.	Tamoxifen, aromatase inhibitors	Effective in hormone receptor-positive cancers; Side effects include hot flashes and increased risk of blood clots.	70%–80% [34,75]
Targeted therapy	Use of drugs targeting specific molecules involved in cancer growth, such as HER2 inhibitors.	Trastuzumab, pertuzumab	Effective in HER2-positive cancers; Side effects include heart problems and infusion reactions.	60%–70% [34,79]

### Prognosis and survival outcomes

The prognosis and survival outcomes for male breast cancer patients are influenced by several factors, including the stage at diagnosis, tumor characteristics, and treatment response [82]. Overall, the survival rates for male breast cancer are comparable to those observed in female breast cancer patients when matched for stage and tumor biology [83,84].

According to data from the Surveillance, Epidemiology, and End Results (SEER) program, the 5-year relative survival rate for male breast cancer patients diagnosed between 2005 and 2010 was approximately 88.5% [85]. However, survival rates can vary significantly based on the stage at diagnosis, with early-stage cancers having a more favorable prognosis compared to advanced-stage or metastatic disease [86].

Several influential factors have been identified in prognosis and enhancing survival outcomes in male breast cancer patients. The tumor stage at diagnosis is one of the most critical prognostic indicators, with earlier stages associated with better survival rates [87]. Additionally, the molecular subtype, the expression of hormone receptors (ER and PR), and HER2 status can impact prognosis and guide treatment decisions [88].

Age at diagnosis is another important prognostic factor, with younger male breast cancer patients generally having better outcomes compared to older patients [86]. However, it is essential to note that male breast cancer is typically diagnosed at a later age than female breast cancer, which may contribute to the overall poorer prognosis observed in some studies [83].

Beyond tumor characteristics and patient factors, the type of treatment received can also influence survival outcomes. Patients who receive appropriate and timely treatment—for instance, surgery, chemotherapy, radiation therapy, and targeted therapies—generally have better prognoses than those who do not receive adequate treatment [89].

Furthermore, long-term survivorship and quality of life considerations are essential aspects to consider in the management of male breast cancer patients. Survivors may face unique physical and psychological challenges, such as body image concerns, sexual dysfunction, and the need for ongoing follow-up and surveillance [90,91]. Supportive care services and resources are crucial to address these issues and promote overall well-being.

### Psychosocial and supportive care needs

Addressing the psychosocial and supportive care needs of male breast cancer patients, specific support services such as counseling, peer support groups tailored for men, and online forums provide critical outlets for emotional and psychological well-being [60]. Resources like the Male Breast Cancer Coalition and the American Cancer Society offer guidance and networking opportunities [60,62,69]. Additionally, family and partners provide emotional, physical, and logistical support, often participating in decision-making and caregiving during treatment. International collaborations can potentially enhance clinical research on a larger scale, leading to more robust and diverse clinical trials. Emerging therapies, such as targeted therapies and immunotherapies, along with advancements in genomic profiling, show promise in improving outcomes for male breast cancer patients. These efforts underline the need for continuous innovation and international cooperation to optimize treatment strategies and support systems.

A diagnosis of male breast cancer can have a profound psychological impact on patients and their families, as it challenges traditional gender norms and societal perceptions of masculinity [18,80]. The rarity of the disease and the lack of awareness can further exacerbate the emotional distress experienced by male breast cancer patients [82].

The psychological impact of a male breast cancer diagnosis can manifest in various forms, including anxiety, depression, body image concerns, and issues related to sexuality and intimacy [92]. Men may feel isolated, stigmatized, or uncomfortable discussing their condition due to the perception that breast cancer is a “woman’s disease” [93]. These feelings can lead to delays in seeking medical attention or avoiding treatment altogether, negatively impacting outcomes.

It is essential to acknowledge and address the unique psychosocial challenges faced by male breast cancer patients [94]. Providing access to support services and resources tailored specifically for men can help alleviate some of the emotional burden and facilitate coping strategies [95].

Support groups, either in-person or online, can offer a safe and understanding environment for male breast cancer patients to share their experiences, concerns, and coping mechanisms with others facing similar challenges [96,97]. Additionally, professional counseling and psychotherapy services can help patients navigate the emotional and psychological aspects of their diagnosis and treatment journey [98].

Involving partners, family members, and close friends in the support system can also be beneficial, as they may experience emotional challenges and need guidance on supporting their loved ones best [99]. Educating and empowering caregivers can help create a more supportive and understanding environment for male breast cancer patients [100].

Furthermore, addressing body image concerns and sexual health issues is crucial for male breast cancer patients [101]. Cosmetic or reconstructive surgery options, as well as counseling and support groups, focused on body image and intimacy, can help patients cope with the physical and emotional challenges associated with their diagnosis and treatment [102].

Male breast cancer patients face unique psychosocial challenges that differ significantly from their female counterparts, primarily due to the rarity of the disease and the perception of breast cancer as a predominantly female condition [80]. Such challenges often result in isolation, embarrassment, and reluctance to seek medical help or engage with psychological support services. Issues of masculinity and body image are particularly pronounced, as the physical changes associated with mastectomy, hormone therapy, or chemotherapy may conflict with traditional male gender roles, leading to a diminished sense of self-esteem and concerns about sexual health and intimacy [79,81]. These challenges are often compounded by societal stigma, which can discourage men from openly discussing their diagnosis or seeking emotional support through counseling or support groups.

Furthermore, men may rely heavily on their partners for emotional and psychological support, while women often seek broader social networks. Male breast cancer patients may also face barriers to accessing mental health resources due to cultural norms that emphasize stoicism and downplay emotional vulnerability in men [65]. To address these unique needs, tailored psychosocial interventions, such as male-specific support groups, counseling that addresses issues related to masculinity, and partner-inclusive therapy, are essential. Understanding the cultural and societal factors that influence the psychosocial experiences of male breast cancer patients can further improve support systems and ultimately enhance their quality of life during and after treatment. By providing comprehensive psychosocial and supportive care services, healthcare professionals can help male breast cancer patients and their families navigate the unique challenges they face, ultimately improving their overall well-being and quality of life [80].

### Limitations of the study

The current study has several limitations. First, the rarity of male breast cancer constrains the availability of extensive and representative data, potentially limiting the generalizability of findings. Additionally, much of the data on male breast cancer derives from studies primarily focused on female breast cancer, leading to the adaptation of treatment guidelines and diagnostic protocols that may not fully account for male-specific biological differences. Another limitation is the lack of consideration for routine screening in men, which often results in delayed diagnosis and limits data on early-stage presentations. Furthermore, the study does not include in-depth analyses of psychosocial impacts, such as stigma and mental health challenges, which are uniquely relevant to male breast cancer patients. Finally, due to limited data, the study may not comprehensively address variations in treatment outcomes for men, especially regarding hormonal therapy efficacy. These limitations underscore the need for further research specifically tailored to male breast cancer.

### Future directions and research opportunities

Despite increasing awareness and research efforts surrounding male breast cancer, significant knowledge gaps remain, warranting further investigation to improve understanding, treatment, and patient outcomes. Ongoing research is crucial to advance male-specific treatment strategies, focusing on the disease’s molecular and genetic basis. For example, international collaborative studies like the MERGE consortium, based at Queen’s University Belfast, are analyzing DNA from 5000 men with breast cancer and 10,000 men without it to identify genetic risk factors and enhance knowledge of the disease [103,104]. Similarly, the International Male Breast Cancer Program, led by the European Organization for Research and Treatment of Cancer (EORTC), addresses knowledge gaps through a three-part initiative [105]. The initiatives include a retrospective analysis of clinical data and tumor samples from cases spanning 20 years, a prospective registry of new cases over 30 months, and clinical studies to optimize patient management. Another significant international effort, led by the EORTC and the Translational Breast Cancer Research Consortium (TBCRC), recently completed a retrospective study of 1483 male breast cancer patients and is launching a prospective cohort study to collect DNA and tumor samples for further research [105,106].

Future research should also emphasize the role of hormonal factors—specifically estrogen and testosterone—and their interplay in male breast cancer’s etiology and progression to inform prevention and risk-reduction strategies. Clinical trials tailored exclusively for male patients are critical to assess the efficacy and safety of both existing and novel treatments. Due to the rarity of male breast cancer, such trials face challenges related to sample size, underscoring the importance of multicenter collaborations and innovative trial designs.

Moreover, investigating the long-term effects of treatment on male breast cancer survivors is crucial. Studies focused on survivorship issues, such as quality of life, physical and psychological well-being, and the impact of treatment on fertility and sexual function, can inform the development of comprehensive supportive care programs tailored to the unique needs of male patients [101].

Raising awareness and promoting advocacy efforts are also critical future directions. Increasing public awareness about male breast cancer can help destigmatize the disease, encourage early detection, and foster a supportive environment for affected individuals [107]. Healthcare professional education is equally important to ensure prompt recognition and appropriate management of male breast cancer cases. In recent years, significant advancements in oncology have introduced innovative approaches that hold promise for improving the diagnosis and treatment of male breast cancer. One such advancement is the role of next-generation sequencing (NGS), which allows for comprehensive genomic profiling of tumors. NGS can identify specific genetic mutations, such as BRCA 1/2 and other less common mutations, that may inform personalized treatment strategies, especially in cases resistant to standard therapies [108]. Additionally, liquid biopsies are emerging as a minimally invasive method to monitor tumor progression and detect circulating tumor DNA (ctDNA). This technique provides real-time insights into tumor dynamics, allowing for earlier intervention and potentially improving outcomes for patients with metastatic or recurrent disease [109,110].

Furthermore, clinical trials explicitly targeting male breast cancer are essential to address the unique molecular characteristics of the disease. Ongoing trials investigating the use of Poly (ADP-ribose) polymerase (PARP) inhibitors, such as olaparib, for patients with BRCA mutations, as well as immune checkpoint inhibitors like pembrolizumab in advanced cases, show promise for expanding treatment options [111,112]. These advancements underscore the importance of integrating precision medicine into the management of male breast cancer, offering a more tailored approach to treatment that could significantly improve survival rates and quality of life for affected men. By focusing on these cutting-edge techniques, future research can drive progress in a disease where large-scale clinical data has historically been limited. Establishing dedicated research programs, funding initiatives, and collaborative networks focused on male breast cancer can accelerate the pace of scientific discovery and translate findings into improved patient care. Completed and ongoing clinical trials for treating Male Breast Cancer are shown in Table 5.

**Table 5:** Completed and ongoing clinical trials for male breast cancer

	Phase	Treatment	Status	Detail
TBCRC 030 [113]	Phase 2	Cisplatin + capecitabine in advanced male breast cancer	Completed	Evaluated this chemotherapy combination in advanced/metastatic male breast cancer.
NCT05501704 [114]	Phase 2	Tamoxifen, anastrozole, degarilex, and abemaciclib for male breast cancer	Recruiting	To evaluate how male breast cancer reacts against preoperative treatment with endocrine treatment and which endocrine treatment regimen is most efficient.
NCT01638247 [115]	Phase 2	Tamoxifen vs. aromatase inhibitor in male breast cancer treatment	Completed	The trial assessed the neoadjuvant, adjuvant, or palliative treatment with aromatase inhibitor vs. tamoxifen in male cancer patients.

## Conclusion

Male breast cancer, though rare, is a significant health concern requiring more attention and research. This review highlights its epidemiology, risk factors, clinical presentation, pathology, treatment strategies, prognosis, and psychosocial impacts. Despite its lower incidence compared to female breast cancer, raising awareness and promoting early detection are crucial to improving outcomes. Genetic predisposition, especially BRCA mutations, hormonal imbalances, environmental exposures, and lifestyle factors, contribute to its etiology. Diagnosing male breast cancer involves imaging and biopsy techniques, with tailored research and therapeutic approaches needed due to its unique characteristics. Treatment strategies mirror those for female breast cancer, including surgery, chemotherapy, radiation, hormonal, and targeted therapies. Addressing body image and masculinity issues is essential in managing male patients. Multidisciplinary care and personalized support services are vital for addressing their physical, emotional, and psychosocial needs. Continued research and awareness are crucial to advancing understanding and improving outcomes for male breast cancer patients.

## Data Availability

Data sharing not applicable to this article as no datasets were generated or analyzed during the current study.
